# The emerging role and targetability of the TCA cycle in cancer metabolism

**DOI:** 10.1007/s13238-017-0451-1

**Published:** 2017-07-26

**Authors:** Nicole M. Anderson, Patrick Mucka, Joseph G. Kern, Hui Feng

**Affiliations:** 1 Abramson Family Cancer Research Institute, University of Pennsylvania, Philadelphia, PA 19104-6160 USA; 2 Perelman School of Medicine at the University of Pennsylvania, Philadelphia, PA 19104 USA; 3 Departments of Pharmacology and Medicine, The Center for Cancer Research, Section of Hematology and Medical Oncology, Boston University School of Medicine, Boston, MA 02118 USA; 4 Program in Biomedical Sciences, Boston University School of Medicine, Boston, MA 02118 USA

**Keywords:** glutaminolysis, the TCA cycle, cancer metabolism, glycolysis

## Abstract

The tricarboxylic acid (TCA) cycle is a central route for oxidative phosphorylation in cells, and fulfills their bioenergetic, biosynthetic, and redox balance requirements. Despite early dogma that cancer cells bypass the TCA cycle and primarily utilize aerobic glycolysis, emerging evidence demonstrates that certain cancer cells, especially those with deregulated oncogene and tumor suppressor expression, rely heavily on the TCA cycle for energy production and macromolecule synthesis. As the field progresses, the importance of aberrant TCA cycle function in tumorigenesis and the potentials of applying small molecule inhibitors to perturb the enhanced cycle function for cancer treatment start to evolve. In this review, we summarize current knowledge about the fuels feeding the cycle, effects of oncogenes and tumor suppressors on fuel and cycle usage, common genetic alterations and deregulation of cycle enzymes, and potential therapeutic opportunities for targeting the TCA cycle in cancer cells. With the application of advanced technology and *in vivo* model organism studies, it is our hope that studies of this previously overlooked biochemical hub will provide fresh insights into cancer metabolism and tumorigenesis, subsequently revealing vulnerabilities for therapeutic interventions in various cancer types.

## Introduction

 Cancer is a disease characterized by the accumulation of genetic alterations and gene deregulations, resulting in uncontrolled cell proliferation that demands both increased energy production and macromolecule synthesis. To cope with increased metabolic stress, malignant cells often reprogram their biochemical pathways to enable rapid uptake and breakdown of nutrients, thus contributing to disease transformation, maintenance, and progression (Hanahan and Weinberg, 2011; Ward and Thompson, 2012). The birth of cancer metabolism research extends back to the early 20th century, when Otto Warburg noted the heavy dependence of cancer cells on glycolysis for growth (Warburg et al., 1927). Indeed, various types of cancer cells increase their glucose uptake and preferentially utilize glucose through aerobic glycolysis (Gillies and Gatenby, 2007; Pavlova and Thompson, 2016). This effect was subsequently applied in the clinic for tumor imaging and detection through positron emission tomography scans of radiolabeled glucose analogs (Papathanassiou et al., 2009). These early findings laid the groundwork for a recent revival of interest in cancer metabolism research, which has lead to discoveries showing overactivation and/or rewiring of multiple metabolic pathways in cancer cells. In just the last ten years, the significance of metabolic reprogramming has led to its inclusion with the classic hallmarks of cancer (Hanahan and Weinberg, 2011). Accumulating evidence indicates that exploiting the unique metabolic dependencies of tumor cells represents an exciting new direction of targeted therapy (Pathania et al., 2009; Kishton and Rathmell, 2015).

The tricarboxylic acid (TCA) cycle is a central hub for energy metabolism and macromolecule synthesis and redox balance. The cycle is composed of a series of biochemical reactions occurring in the mitochondrial matrix, which allow aerobic organisms to oxidize fuel sources and provide energy, macromolecules, and redox balance to the cell. Aberrant TCA cycle function is implicated in a wide variety of pathological processes. Genetic diseases with compromised TCA cycle function due to inherited cycle enzyme mutations, such as fumarase (FH) deficiency, are rare but severe (Rustin et al., 1997). Moreover, several TCA cycle enzymes are deregulated in obesity, including citrate synthase, which exhibits reduced activity in obese mice (Cummins et al., 2014). Multiple neurodegenerative disorders such as Alzheimer’s disease are associated with reduced activity of the α-ketoglutarate dehydrogenase complex (KGDHC) (Gibson et al., 2010). In light of the widely accepted belief that cancer cells primarily utilize aerobic glycolysis, the role of the TCA cycle in cancer metabolism and tumorigenesis has been overlooked until recently.

With the application of contemporary technology, such as unbiased and targeted metabolomics, as well as genetic and biochemical studies using animal models, many recent advances have been made in the field of cancer metabolism. Studies have demonstrated that tumor cells can indeed uncouple glycolysis from the TCA cycle, allowing the use of additional fuel sources such as glutamine to meet their heightened metabolic needs (Chen and Russo, 2012) (Pavlova and Thompson, 2016). Importantly, glutamine is now established as an important nutrient source across numerous cancer types, especially for MYC-driven cancers (DeBerardinis and Cheng, 2010). The role of lipid metabolism in tumorigenesis has also received increased attention in recent years. Altogether, these studies have provided convincing evidence to establish the role of the TCA cycle in cancer metabolism and tumorigenesis (Sajnani et al., 2017). Importantly, various oncogenes and tumor suppressors regulate both the uptake and breakdown of fuel sources in the TCA cycle by regulating the expression of fuel transporters and/or activity of cycle enzymes in cancer cells (Chen and Russo, 2012). Multiple cycle enzymes, including aconitase (also known as aconitate hydratase, AH), isocitrate dehydrogenase (IDH), FH, succinate dehydrogenase (SDH) and KGDHC, are frequently mutated or deregulated in human cancers (Eng et al., 2003; Juang, 2004; Yan et al., 2009). Recent results from clinical testing suggest that targeting reprogrammed metabolic pathways, including the TCA cycle, could provide a new and promising therapeutic avenue for the treatment of a broad spectrum of cancers.

## Fuels feeding the TCA cycle

The TCA cycle serves as a convergence point in the cellular respiration machinery, which integrates multiple fuel sources derived from the diet including glucose, glutamine, and fatty acids. Through various biochemical reactions, the cycle produces intermediates for use as building blocks in macromolecule synthesis, as well as energy and electron acceptors that are utilized in downstream cellular processes such as the electron transport chain (ETC) reactions. Although both normal and tumor cells can catabolize all major types of fuels, they differ in the rate of uptake and catabolism of each fuel. While glucose provides the main source of pyruvate entering the TCA cycle in normal cells, cancer cells often shunt glucose away from the TCA cycle for catabolism through anaerobic glycolysis, and thus are more dependent on glutamine and fatty acids to replenish TCA cycle intermediates (Eagle, 1955).

### Glucose

**Figure 1:**
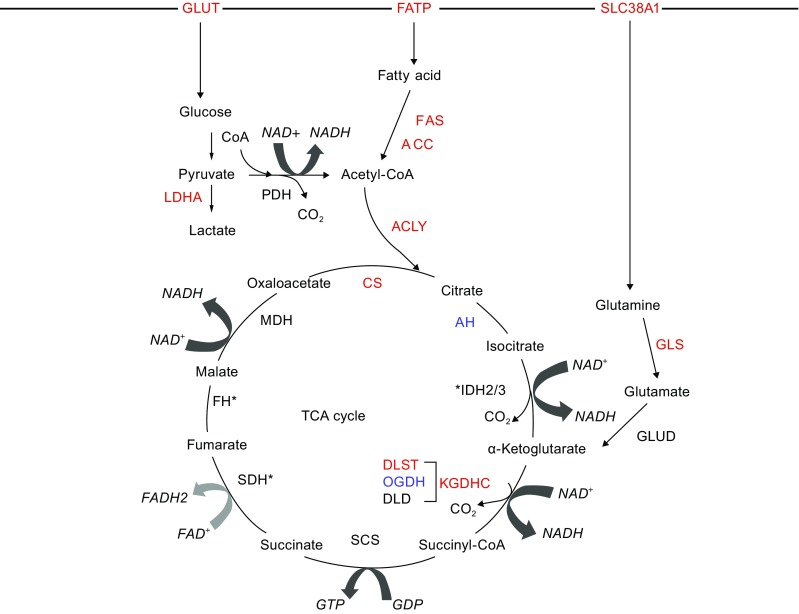
**Transporters, fuels, enzymes, and biochemical reactions driving the TCA cycle**. The typical input for the TCA cycle is acetyl-CoA, which is derived from pyruvate, the end product of glycolysis. Through a series of redox reactions, chemical bond energy from acetyl-CoA is harvested to produce high-energy electrons, which are carried to the electron transport chain by nicotinamide adenine dinucleotide (NAD^+^) and flavin adenine dinucleotide (FAD). Subsequent oxidative phosphorylation results in the production of adenosine triphosphate (ATP) from each acetyl-CoA. Because oxygen is required to regenerate NAD^+^ and FAD, the TCA cycle only proceeds in aerobic environments. There are a total of 8 steps in the TCA cycle, three of which are irreversible; the generation of citrate from oxaloacetate and acetyl-CoA by CS; the conversion of isocitrate to α-KG by IDH3; and the formation of succinyl-CoA from α-KG by KGDHC (Berg JM, 2002; Akram, 2014). The biochemical reactions in the TCA cycle are regulated by several means including substrate availability, product inhibition, and allosteric regulation, allowing the cell to control energy production based on its energy status (NADH/NAD^+^ ratio, ATP availability) and nutrient availability (Berg JM, 2002). Intermediates in the cycle can be derived from outside sources, such as the production of acetyl-CoA from β-oxidation of fatty acids or the production of α-KG from protein catabolism, particularly glutaminolysis (Houten and Wanders, 2010; Akram, 2014). Importantly, deregulation of TCA cycle enzymes, such as mutations and gene deregulations, or aberrant accumulation of TCA intermediates can have disease-relevant consequences. Proteins that are upregulated in cancer are highlighted as red and downregulated as blue, while enzymes mutated are marked with an asterisk. Abbreviations: CS: citrate synthase, AH: aconitase, IDH: isocitrate dehydrogenase, KGDHC: α-ketoglutarate dehydrogenase complex, OGDH: α-KG dehydrogenase, DLST: dihydrolipoamide S-succinyltransferase, DLD: dihydrolipoamide dehydrogenase, SCS: succinyl-CoA synthase, SDH: succinate dehydrogenase, FH: fumarate hydratase, MDH: malate dehydrogenase, PDH: pyruvate dehydrogenase, GLUT: glucose transporter, FATP: fatty acid transporter, SCL38A: sodium-coupled neutral amino acid transporter, ACLY: adenosine triphosphate citrate lyase, ACC: acetyl-CoA carboxylase, FAS: fatty acid synthase, GLS: glutaminase, GDH: glutamate dehydrogenase

Glucose is imported into the cell by glucose transporters (GLUT) and serves as the most common fuel source in mammalian cells (Fig. 1). In normal cells, most cellular glucose enters the TCA cycle in the form of pyruvate, although glucose can also be utilized for lactate production or macromolecular synthesis through the pentose phosphate pathway (Fig. 1). Through glycolysis, one glucose molecule is converted into two pyruvate molecules, which are primarily oxidized to produce acetyl-CoA feeding the TCA cycle. Alternatively, under hypoxic conditions, pyruvate may be converted to lactate as well. Progression through the TCA cycle occurs when heightened energetic needs arise (Fig. 1). Glucose can also be synthesized through gluconeogenesis, a process reciprocally regulated compared to glycolysis in order to keep the metabolism of the cell efficient (Berg JM, 2002).

Cancer cells markedly increase their glucose usage, as noted by Otto Warburg nearly 100 years ago. Tumors acquire additional glucose by upregulating the high-affinity glucose transporters GLUT1 and GLUT3, while simultaneously downregulating lower affinity transporters (Birnbaum et al., 1987; Baron-Delage et al., 1996). Not only do cancer cells increase the rate of glucose uptake and utilization, but the fate of imported glucose differs from that in normal cells as well. While normal cells and some cancer cells, such as lung cancer stem cells and leukemic cells, oxidize glucose in the mitochondria (Gatenby and Gillies, 2004; Gao et al., 2016; Kishton et al., 2016), most cancer cells preferentially break down glucose to produce lactate even in normoxic conditions (Kim et al., 2006), The process of aerobic glycolysis only generates 2 ATP per glucose molecule, a drastic reduction from 38 ATP when glucose is oxidized through the TCA cycle. To meet their heightened energetic needs, cancer cells turns to other fuel sources, such as glutamine, to feed the TCA cycle.

### Glutamine

In addition to glucose, amino acids can also fuel the TCA cycle. Amino acids enter the cycle after being converted to either acetyl-CoA or α-keto acid intermediates: pyruvate, oxaloacetate, and succinyl-CoA (Berg JM, 2002). Glutamine is the most abundant amino acid in the human body, serving to transport nitrogen in the plasma for biosynthesis of non-essential amino acids, such as purines and pyrimidines, as well as fatty acids, or entering the TCA cycle in the form of α-ketoglutarate (α-KG) (Reitzer et al., 1979; Brosnan, 2003; Wang et al., 2017). Glutaminolysis, the breakdown of glutamine, is critical in replenishing cycle intermediates in proliferating cells. Glutamine is first hydrolyzed by glutaminase (GLS) to yield glutamate, which subsequently is either dehydrogenated by glutamate dehydrogenase (GLUD) to form α-KG or functions as a co-substrate for the transaminases, glutamate oxaloacetate transaminase and glutamate pyruvate transaminase to form alanine and aspartate respectively. α-KG is a substrate for oxidative decarboxylation by KGDHC or for reductive carboxylation by IDH2 (Mullen et al., 2011). Thus, glutaminolysis serves as a common pathway for both anaplerotic and cataplerotic processes.

The importance of glutaminolysis in cancer cell proliferation was noted decades ago by Harry Eagle, who found that HeLa cells preferred a molar excess of 10- to 100- fold of glutamine for maximum growth (Eagle, 1955). This metabolic dependence is partially driven by the glycolytic phenotype seen in certain types of cancer cells. Due to the excessive conversion of glucose to lactate, tumor cells use anaplerotic reactions to replenish TCA cycle intermediates, which is largely achieved through increased glutaminolysis (DeBerardinis et al., 2007). To do so, cancer cells upregulate both glutamine transporters and enzymes catalyzing glutaminolysis, thus uncoupling this pathway from growth factor-mediated stimuli (Fig. 1) (Pavlova and Thompson, 2016). The proto-oncogene *MYC* is a critical regulator of glutaminolysis and upregulates both glutamine transporters and GLS (Wise et al., 2008; Gao et al., 2009). Elevated levels of GLS and glutamine transporters enable tumor cells to derive large portions of their energy and macromolecules through glutamine catabolism, leading to glutamine addiction in numerous cancer types including myeloma and glioma (Bolzoni et al., 2016; Márquez et al., 2017).

### Fatty acids

The third type of fuel source in cancer cells is fatty acids, which enter the TCA cycle after undergoing β-oxidation to generate acetyl-CoA. Acetyl-CoA is the substrate for both the fatty acid synthesis pathway and the TCA cycle, making lipogenesis an important convergence point for TCA cycle flux and cellular biosynthesis (Migita et al., 2008). In the process of β-oxidation, the acyl chain undergoes oxidation, introducing a double bond, followed by hydration to alcohol and oxidation to ketone. Finally, co-enzyme A cleaves the acyl tail to yield an acetyl-CoA and reduces the fatty acid chain length by two carbons. This process generates more acetyl-CoA per molecule than does either glucose or glutamine (Berg JM, 2002). *De novo* synthesis of fatty acids is critical to supply lipids for cell membrane formation in rapidly proliferating cells, and is regulated by fatty acid biosynthetic enzymes: adenosine triphosphate citrate lyase (ACLY), acetyl-CoA carboxylase (ACC), and fatty acid synthase (FAS). ACLY converts citrate to oxaloacetate and cytosolic acetyl-CoA. This cytosolic acetyl-CoA is carboxylated by ACC to form malonyl-CoA, which is then combined with additional acetyl-CoA until the 16-carbon unsaturated fatty acid palmitate is formed. Palmitate can then be modified to form additional required components of cell membrane.

While enzymes regulating lipid synthesis are often expressed in low levels in most normal tissue (Clarke, 1993), they are overexpressed in multiple types of cancers. ACLY is overexpressed in non-small cell lung cancer, breast cancer, and cervical cancer among others (Migita et al., 2008; Xin et al., 2016; Wang et al., 2017). ACC is upregulated in non-small cell lung cancer and hepatocellular carcinoma (Wang et al., 2016; Svensson and Shaw, 2017). FAS is overexpressed in prostate and breast cancers (Swinnen et al., 2002; Menendez et al., 2004). In tumor cells where the demand is much greater, lipogenesis occurs via these overexpressed enzymes. The increased activation and overexpression of these enzymes in tumors correlates with disease progression, poor prognosis, and is being investigated as a potential biomarker of metastasis (Xin et al., 2016).

## Oncogenes and tumor suppressors impinging on the TCA cycle

Genetic alterations and/or deregulations of tumor suppressors or oncogenes often drive metabolic reprograming in cancers, although this effect can differ based on specific alterations or deregulations, and is often context-dependent. Several oncogenes, including *MYC*, *HIF*, *P53*, and *RAS*, are known to regulate the metabolic phenotype of tumors and play a critical role in determining how the TCA cycle is utilized in these cancer cells.

### MYC

The proto-oncogene *MYC* controls a wide range of cellular processes, including cell proliferation, metabolism, cellular differentiation and genomic instability, and is a dominant driver of tumor transformation and progression (Meyer and Penn, 2008). Aberrant MYC activity, resulting from chromosomal translocations, gene amplifications or increased mRNA/protein stability, is found in over half of all human cancers (Gabay et al., 2014). Importantly, MYC is a central regulator of cellular metabolism, and can promote a broad range of metabolic pathways, such as aerobic glycolysis, glutaminolysis, mitochondrial biogenesis, oxidative phosphorylation, and nucleotide and amino acid biosynthesis (Adhikary and Eilers, 2005; Gabay et al., 2014; Wahlstrom and Henriksson, 2015). As stated early in this review article, MYC transcriptionally activates key genes and enzymes regulating glutaminolysis, and serves as the principal driver of glutamine metabolism through the TCA cycle (i.e., glutamine anaplerosis). Specifically, to promote the import of glutamine into the cell, MYC transcriptionally upregulates glutamine transporters ASC amino acid transporter 2 (ASCT2) and system N transporter (SN2). Additionally, Gao et al. demonstrated that MYC controls the conversion of glutamine to glutamate by activating glutaminase 1 (GLS1) through transcriptional suppression of its negative regulator miR-23a/b (Wise et al., 2008; Gao et al., 2009). There are two independent pathways that control the conversion of glutamate to α-KG entering the TCA cycle: one controlled by GLUD and another by aminotransferases. MYC-dependent cancer cells can utilize either GLUD or aminotransferases to convert glutamine to α-KG for the TCA cycle (Wise et al., 2008; Wang et al., 2011). MYC may also play a role in directing fatty acid oxidation and directing its metabolites into the TCA cycle by way of acetyl-CoA. Specifically, MYC expression leads to the upregulation of fatty acid transporters (e.g., fatty acid-binding protein 4) and fatty acid oxidation genes such as hydroxyacyl-CoA dehydrogenase (Wang et al., 2011; Edmunds et al., 2015).

### HIF

Hypoxia-inducible factors (HIFs) are transcription factors that respond to reduced oxygen availability. HIFs are heterodimers composed of an oxygen-dependent α-subunit and a constitutively expressed β-subunit. Under normoxia, the α-subunit is targeted for degradation upon hydroxylation by prolyl hydroxylases (PHD) and subsequent ubiquitination by von Hippel-Lindau (VHL) tumor suppressor. Tumors activate HIFα either in the face of hypoxia resulting from poor vascularization or due to genetic abrogation such as *VHL* loss (Gordan and Simon, 2007). HIF activation orchestrates a metabolic program that promotes the catabolism of glucose through aerobic glycolysis and thus shifts glucose away from the TCA cycle (Semenza, 2012). HIF promotes glycolysis and lactate production through transcriptional upregulation of glucose transporters (SLC2A1 and SLC2A3), glycolytic enzymes (e.g., hexokinase (HK) and pyruvate kinase (PK)), and lactate dehydrogenase A (LDHA) (Kim et al., 2006). Kim et al. demonstrated that HIF1 suppresses glucose metabolism through the TCA cycle (i.e., glucose anaplerosis) by directly activating pyruvate dehydrogenase kinase 1 (PDK-1), a negative regulator of cycle enzyme pyruvate dehydrogenase (PDH) (Kim et al., 2006). To compensate for the reduction of glucose feeding the TCA cycle, tumor cells with HIF activation often increase the usage of glutamine (Le et al., 2012). Under hypoxia conditions, glutamine largely fuels the TCA cycle in the form of α-KG to promote reductive carboxylation that produces citrate for lipogenesis (Wise et al., 2008; Metallo et al., 2011; Gameiro et al., 2013).

### P53

P53 is a transcription factor and known tumor suppressor that regulates many important cellular pathways, including cell survival, DNA repair, apoptosis, and senescence (Bensaad et al., 2009). Wild-type P53 plays an important role in metabolism by striking a balance between bioenergetics and biosynthesis. One of the ways it does so is by lowering rates of glycolysis and promoting oxidative phosphorylation. P53 acts to suppress glycolysis by directly downregulating glucose transporters (GLUT1 and GLUT4) and indirectly inhibiting the activity of glycolytic enzymes, phosphofructokinase 1 (PFK1) and phosphoglycerate mutase (Kondoh et al., 2005; Bensaad et al., 2006, 2009; Zhang et al., 2013). To promote oxidative phosphorylation, P53 ensures availability of anapleurotic substrates, glucose, and glutamine, to the TCA cycle. As an activator of PDH, P53 downregulates PDH’s negative regulator PDK2 and indirectly activates PDHA1 (PDH A1 subunit). Additionally, P53 promotes glutamine incorporation into the TCA through direct transcriptional upregulation of glutaminase 2 (GLS2) (Zhang et al., 2011; Contractor and Harris, 2012). In solid tumors, *P53* is commonly mutated and somatic mutations of *P53* occur in more than 50% of human malignancies (Kruiswijk et al., 2015). Subsequently, loss of wild-type P53 function has a significant impact on cellular metabolism, leading to enhanced glycolysis and repressed oxidative phosphorylation in these tumor cells.

### RAS

The most frequently mutated *RAS* subfamily genes in cancer are *KRAS*, *NRAS*, and *HRAS*, which serve as intercellular signaling molecules to transduce extracellular signaling from receptor tyrosine kinase to downstream effectors (Pylayeva-Gupta et al., 2011; Stephen et al., 2014). RAS plays a critical role in activating scavenging pathways in certain types of tumors and promotes nutrient uptake through both the extracellular and intracellular sources (Pylayeva-Gupta et al., 2011; Stephen et al., 2014). For example, Kamphorst et al. demonstrated that *KRAS*-driven pancreatic cells scavenge proteins, such as glutamine, from the extracellular space and utilize them to fuel the TCA cycle (Kamphorst et al., 2015). Additionally, it has been shown that *KRAS*-driven non-small cell lung cancer cells utilize autophagy to access intracellular supplies of glutamine to promote TCA cycle function (Guo et al., 2011; Strohecker and White, 2014). Moreover, *KRAS*-driven cancer cells can scavenge branch chain amino acids (i.e., isoleucine, valine, and leucine) and convert them into acetyl-CoA to fuel the TCA cycle (Mayers et al., 2014). A recent study by Kerr et al. demonstrated that copy number gain of mutant *KRAS* associated with tumor progression can promote glucose anaplerosis to fuel the TCA cycle (Kerr et al., 2016).

## Cycle enzyme alterations in cancer

The biochemical reactions in the TCA cycle are catalyzed by a number of enzymes. Recent findings show that multiple cycle enzymes are either mutated or deregulated in a broad spectrum of cancer, resulting in characteristic metabolic and epigenetic changes that are correlated with disease transformation and progression.

### SDH

Succinate dehydrogenase (SDH), also known as complex II, has roles in the TCA cycle and the ETC. SDH is a heterotetrameric enzyme complex composed of 4 subunits (SDHA, SDHB, SDHC, and SDHD), which catalyzes the oxidation of succinate to fumarate in the TCA cycle, while simultaneously reducing ubiquinone to ubiquinol in the ETC (Chandel, 2015). Mutations in *SDHA*, *SDHB*, *SDHC*, *SDHD* and SDH assembly factor 2 (*SDHAF2*) have been identified in hereditary paragangliomas (hPGLs) and pheochromocytomas (PCCs) (Table 1) (Baysal et al., 2000; Niemann and Muller, 2000; Astuti et al., 2001; Baysal et al., 2002; Hao et al., 2009; Bayley et al., 2010; Burnichon et al., 2010). Heterozygous mutations in *SDH* predispose patients to hPGL and PCC. Loss of heterozygosity as a result of a second mutation in the wild-type *SDH* allele triggers neoplastic transformation; thus, *SDH* is classified as a tumor suppressor gene (Gottlieb and Tomlinson, 2005). Additionally, mutations in *SDH* have also been identified in gastrointestinal stromal tumors, renal tumors, thyroid tumors, neuroblastoma, and testicular seminoma, implicating its importance in a wide range of cancer (Bardella et al., 2011).

**Table 1:** Summary of cycle enzyme genetic alterations in cancer

Gene	Genetic alterations	Tumor context	Consequence of alterations	References
*SDHA*	c.91C>T c.1765C>T c.212G>A c.674C>T, c.818C>T c.341A>G c.367C>A c.441delG c.725_736del c.989_9990insTA c.1753C>T c.1865G>A c.1873C>T c.1886A>T	Paragangliomas Pheochromocytomas	Leads to reduction or loss of enzymatic activity of the SDH catalytic subunit and defective function of mitochondrial complex II	(Burnichon et al., 2010; Bardella et al., 2011; Korpershoek et al., 2011; Dwight et al., 2013; Evenepoel et al., 2015; Pillai et al., 2017)
	c.2T>C c.91C>T c.113A>T c.160C>T c. 206C>T c.224G>A c.244A>T c.T273I c.457-3 457-1delCAG c.457-2 c457delCAG c.511C>T c.553C>T c.562C>T c.688delG c.767C>T c.778G>A c.800C>T c.818C>T c.985C>T c.1043-1055del c.1046 147delTG c.1151C>G c.1255G>A c.1334C>T c.1357G>A c.1361C>A c.1471G>T c.1534C>T c.1690G>A c.1765C>T c.1766G>A c.1794G>C c.1795-1G>T c.1873C>T c.1969G>A	Gastrointestinal stromal tumors		(Pantaleo et al., 2011; Italiano et al., 2012; Belinsky et al., 2013a; Belinsky et al., 2013b; Miettinen et al., 2013; Oudijk et al., 2013; Miettinen and Lasota, 2014; Evenepoel et al., 2015; Jiang et al., 2015)
	c.2T>C	Renal cell carcinoma		(Jiang et al., 2015)
*SDHB*	c.-1- ?_72+ ?del c.-1- ?_765+ ?del c.3G>A c.21delC c.49delA c.72+1G>A c.73_76delGCCT c.79C>A c.136C>G c.137G>A c.141G>A c.155delC c.166-170delCCTCA c.203G>A c.213C>T c.221insCCAG c.238A>G c.268C>T c.269G>A c.270C>G c.271G>A c.277T>C c.287-2A>G c.287- ?_540+ ?del c.291G>A c.293G>A c.299C>T c.300-4delCCTCA c.300T>C c.312insCACTGCA c.328C>T c.394T>C c.402C>T c.416T>C c.421-2A>G c.423+1G>A c.438G>A c.540G>A c.541-2A>G c.549_552delTACinsATACAG c.557G>A c.558-3C>G c.566G>A c.589C>T c.649C>T c.650G>T c.653G>A c.688C>T c.689G>A c.689G>T c.708T>C c.718_719delCT c.721G>A c.724C>G c.724C>A c.724C>T c.725G>A c.736A>T c.761C>T c.765+1G>A c.778G>C c.780delG c.847delTCTC c.859G>A c.881C>A c.889+1G>A	Paraganglioma Pheochromocytoma	Reduces SDH catalytic activity and causes defects in enzymatic activity in mitochondrial complex II	(Neumann et al., 2004, 2009; Bardella et al., 2011; Sjursen et al., 2013; Evenepoel et al., 2015; Bennedbaek et al., 2016)
	c.32G>A c.88delC c.136C>T c.137G>A c.847-50delTCTC	Renal cell carcinoma		(Vanharanta et al., 2004; Ricketts et al., 2008; Paik et al., 2014)
	c.392delC	Thyroid carcinoma		(Zantour et al., 2004)
	IVS1+1G>T c.17_dup26GTCG{dup26}GCCA c.17 42dup c.43+1C>T c.45_46insCC c.72+1G>T c.137G>A c.274T>A c.380T>G c.423+1G>C c.423+1G>A c.423+20T>A c.600G>T c.725G>A	Gastrointestinal stromal tumors		(McWhinney et al., 2007; Pasini et al., 2008; Janeway et al., 2011; Miettinen et al., 2013; Miettinen and Lasota, 2014)
	c.418G>T	Neuroblastoma		(Schimke et al., 2010)
	c.587G>A	Pituitary carcinoma		(Tufton et al., 2017)
	c.136C>T	T-cell acute leukemia		(Baysal, 2007)
*SDHC*	c.1A>G c.2T>A c.3G>A c.23dupA c.39C>A c.43C>T c.77 + 4760A>G c.78-2A>G c.78-19C>T c.112A>G c.126G>A c.140-5527C>A c.148C>T c.166A>T c.173T>C c.191_207del17 c.210C>G c.214C>T c.218insA c.224G>A c.242G>T c.242-5580C>A, c.212C>A c.253_255dupTTT c.397C>T c.405+1G>T c.439C>T c.496C>G IVS4+1G>A	Paraganglioma Pheochromocytoma	Leads to reduced SDH enzymatic activity and defective function in mitochondrial complex II	(Douwes Dekker et al., 2003; Mannelli et al., 2007; Peczkowska et al., 2008; Neumann et al., 2009; Bennedbaek et al., 2016; Pillai et al., 2017)
	IVS5+1G>A c.1A>G c.6delT c.43C>T c.57delG c.224G>A c.301delT c.380A>G c.397C>T c.405+1G>A c.455G>C	Gastrointestinal stromal tumors		(McWhinney et al., 2007; Pasini et al., 2008; Janeway et al., 2011; Miettinen et al., 2013; Miettinen and Lasota, 2014)
*SDHD*	c.2T>A c.3G>C c.14G>A c.33C>A c.33C>T c.34G>A c.36_37delTG c.49C>T c.50G>T c.52+2T>G c.53-2A>G c.53+2T>G c.55dupT c.64C>T c.106C>T c.112C>T c.118A>G c.120_ 127delCCCAGAAT c.129G>A c.149A>G c.168_169delTT c.169 + 5G>A, c.53-889G>A c.170-1G>T c.184_185insTC c.191_192delTC c.204-216del13bp c.206_218del13bp c.208A>G c.230T>G c.233_242del10bp c.242C>T c.252T>G c.274G>T c.276_278delCTA c.284T>C c.302T>C c.314+1G>C c.317delG c.325C>T c.334_337delACTG c.337_340delGACT c.341A>G c.341_342delAT c.361C>T c.367G>A c.370delG c.386_387insT c.408delT c.416T>C c.441delG c.443G>T IVS1+2T>G	Paraganglioma Pheochromocytoma	Reduces efficacy of SDH and impairs mitochondrial complex II activity	(Gimm et al., 2000; Taschner et al., 2001; Dannenberg et al., 2002; Douwes Dekker et al., 2003; Lee et al., 2003; Neumann et al., 2004; Simi et al., 2005; Galera-Ruiz et al., 2008; Neumann et al., 2009; Evenepoel et al., 2015; Bennedbaek et al., 2016; Pillai et al., 2017)
	c.34G>A c.57delG c.352delG c.416T>C	Gastrointestinal stromal tumors		(Pasini et al., 2008; Janeway et al., 2011; Miettinen et al., 2013; Oudijk et al., 2013; Miettinen and Lasota, 2014)
	c.129G>A	Testicular seminoma		(Galera-Ruiz et al., 2008; Evenepoel et al., 2015)
*SDHAF2*	c.68C>T c.139A>G c.232 G>A	Paraganglioma Pheochromocytoma	Leads to loss of flavination of SDH, reducing stability and activity of the enzyme complex	(Hao et al., 2009; Bayley et al., 2010; Pillai et al., 2017)
*FH*	p.Gln4X 1-bp del. In codon 17 p.Arg58X p.Asn64Thr p.Ala74Pro p.His137Arg p.Gln142Arg 2-bp del. In codon 181 Lys187Arg Lys del. In codon 187 Arg190His -15 splice site p.Gly239Val p.Arg300X 1-bp del. In codon 507	Multiple leiomyomatosis	Leads to loss of FH enzymatic activity and accumulation of fumarate in the cell	(Tomlinson et al., 2002)
	c.1?_c.*100del c.1?_404+?del c.111insA c.127_128delGA c.138+1_138+10del10 c.147delT c.157G>T c.172C>T c.191A>C c.220G>C c.233del c.247_249+1delGAGGinsA c.250-2A>G c.266T>C c.298delA c.305C>G c.349A>G c.410A>G c.425A>G c.431C>T c.434A>G c.455T>C c.503T>C c.560A>G c.568C>T c.568delAC c.569G>A c.569G>T c.575A>G c.632A>G c.666delC c.698G>A c.780delGC c.782-788 7-bp del. c.806T>C c.808G>T c.810delA c.815T>C c.821C>T c.823C>T c.824G>A c.836T>A c.869G>A c.875T>C c.891T>A c.898C>T c.952C>T c.964A>G c.968G>A c.989A>G c.1002T>G 2-bp ins @1004 c.1020T>A c.1025C>A c.1028A>G c.1060G>A c.1083-1086delTGAA c.1108-2A>G c.1121-1123 del TAC c.1123delA c.1126T>C c.1138insA c.1144A>G c.1162delA c.1187A>C c.1189G>A c.1210G>T c.1234del c.1265A>G 8-bp dup @ 1300-1307 c.1339delG c.1349-1352delATGA c.1371G>A c.1431insAAA	Hereditary leiomymatosis and renal cell carcinoma		(Toro et al., 2003; Wei et al., 2006; Pfaffenroth and Linehan, 2008; Gardie et al., 2011; Smit et al., 2011; Chen et al., 2014; Wong et al., 2014; Arenas Valencia et al., 2017)
	c.220G>C c.426+1G>A c.988A>G c.994delA	Type 2 papillary renal cell carcinoma		(Gardie et al., 2011)
	c.1394G>A c.352A>C	Leydig cell tumors (Carvajal-Carmona et al.)		(Carvajal-Carmona et al., 2006)
	435insAAA 691G>A	Ovarian mucinous cystadenoma		(Ylisaukko-oja et al., 2006)
*IDH1*	p.Arg100Gln p.Arg132His p.Arg132Cys p.Arg132Ser p.Arg132Leu p.Arg132Gly	Gliomas/Glioblastomas	Increases affinity for NADPH/α-KG; reduces affinity for isocitrate; increases production of 2-HG	(Parsons et al., 2008; Dang et al., 2009; Yan et al., 2009; Pusch et al., 2011)
	p.Arg132His p.Arg132Cys p.Arg132Ser p.Arg132Gly p.Arg132Leu	Acute myeloid leukemia		(Mardis et al., 2009; Abbas et al., 2010; Bayley et al., 2010)
	p.Arg132Cys p.Arg132Leu p.Arg132Gly p.Arg132Ser	Myelodysplastic syndromes/ Myeloproliferative neoplasms		(Kosmider et al., 2010; Pardanani et al., 2010)
	p.Arg132Cys p.Arg132His p.Arg132Leu p.Arg132Ser	Chondrosarcoma		(Amary et al., 2011)
	p.Arg132His p.Arg132Gly p.Arg132Ser p.Arg132Cys	Acute lymphoblastic leukemia		(Kang et al., 2009; Zhang et al., 2012)
	p.Gly70Asp p.Val71Ile p.Gly105Gly; p.Val1781Ile p.Gly123Arg p.Ile130Met p.His133Gln p.Ala134Asp	Thyroid carcinoma		(Hemerly et al., 2010; Murugan et al., 2010)
	p.Arg132Cys p.Arg132His	Prostate carcinoma		(Kang et al., 2009; Ghiam et al., 2012)
*IDH2*	p.Arg172Gly p.Arg172Met p.Arg172Lys	Gliomas/Glioblastomas	Increases affinity for NADPH/α-KG; reduces affinity for isocitrate; increases production of 2-HG	(Yan et al., 2009)
	p.Arg140Gln p.Arg172Lys p.Arg172Gln p.Arg172Thr p.Arg172Gly	Angioimmunoblastic T-cell lymphoma		(Cairns et al., 2012; Lemonnier et al., 2016)
	p.Arg140Gln p.Arg140Trp p.Arg172Lys p.Arg172Met	Acute myeloid leukemia		(Abbas et al., 2010; Gross et al., 2010; Pardanani et al., 2010)
	p.Arg140Gln p.Arg140Leu	Myelodysplastic syndromes/ Myeloproliferative neoplasms		(Kosmider et al., 2010; Pardanani et al., 2010)
	p.Arg172Ser	Chondrosarcoma		(Amary et al., 2011)

### FH

Fumarate hydratase (FH) is a homotetrameric cycle enzyme that catalyzes the stereospecific and reversible hydration of fumarate to L-malate. Beyond its mitochondrial role, *FH* is also expressed in the cytoplasm where it participates in the urea cycle as well as nucleotide and amino acid metabolism (Adam, 2014 #160). Heterozygous mutations in *FH* predispose patients to multiple cutaneous and uterine leiomyomas (MCUL), as well as hereditary leiomyomatosis and renal cell cancer (HLRCC) (Table 1) (Launonen et al., 2001; Tomlinson et al., 2002). Additionally, mutations in FH have been identified in bladder, breast and testicular cancer (Table 1) (Carvajal-Carmona et al., 2006; Ylisaukko-oja et al., 2006). Mutations predisposing patients to MCUL or HLRCC occur across the gene and include missense, frameshift, nonsense and large deletions at the *FH* locus (Table 1) (Bensaad et al., 2006). Similar to SDH, the enzymatic activity of FH is completely absent in HLRCC patients due to a loss of the remaining wild-type allele (Wei et al., 2006).

### IDH

The IDH family is comprised of three isoforms (IDH1, IDH2, and IDH3) that convert isocitrate to α-KG. Only *IDH2* and *3* are expressed in the mitochondria, while *IDH1* is expressed in the cytoplasm. IDH1 and IDH2 function as homodimers that catalyze the conversion of α-KG to isocitrate and require NADP^+^ as a co-factor, whereas IDH3 is a heterodimer (IDH3A, IDH3B, and IDH3G) that can only oxidize isocitrate to α-KG and requires NAD^+^ as a co-factor (Chandel, 2015). Unlike *FH* and *SDH*, mutations in *IDH1* and *2* are somatic heterozygous missense mutations that occur primarily at the active arginine residues that are critical for isocitrate binding (IDH1: R132; IDH2: R172, R140; Table 1) (Parsons et al., 2008; Dang et al., 2009; Mardis et al., 2009; Yan et al., 2009). No mutations in *IDH3* have been reported so far. IDH1/2 mutations occur frequently in low-grade glioma and secondary glioblastoma (~80%), but can also occur in acute myeloid leukemia (20%), angioimmunoblastic T-cell lymphomas (20%), and rarely in other malignancies such as thyroid, colorectal, and prostate cancer (Table 1) (Kang et al., 2009; Yen et al., 2010; Ghiam et al., 2012; Ohgaki and Kleihues, 2013; Yen et al., 2017). These neomorphic mutations result in the gained function of converting α-KG to 2-hydroxyglutarate (2-HG), an oncometabolite.

### Deregulation of other cycle enzymes

Beyond mutations detected for cycle enzymes, several studies have demonstrated that other cycle enzymes, CS, AH, and KGDHC, are deregulated in cancer. CS catalyzes a rate-limiting step in the TCA cycle and is either overexpressed or has increased enzymatic activity in pancreatic, ovarian, and renal cancer (Schlichtholz et al., 2005; Lin et al., 2012; Chen et al., 2014). AH is a reversible enzyme that catalyzes the conversion of citrate to isocitrate and its expression is downregulated in both gastric and prostate cancer (Singh et al., 2006; Wang et al., 2013). KGDHC is a rate-limiting enzyme of the TCA cycle and has three components including α-KG dehydrogenase (OGDH), dihydrolipoamide S-succinyltransferase (DLST), and dihydrolipoamide dehydrogenase (DLD). OGDH is downregulated in colorectal cancer as the result of promoter hypermethylation and similar promoter hypermethylation has been documented in breast, lung, esophageal, cervical, and pancreatic cancer (Hoque et al., 2008; Ostrow et al., 2009; Fedorova et al., 2015). Interestingly, Snezhkina and colleagues have demonstrated that an alternative splice variant of *OGDH* that is tumor specific is overexpressed in colorectal cancer (Snezhkina et al., 2016). OGDH is regulated by Ca^2+^, adenine nucleotides, and NADH, and the tumor-specific isoform lacks three regions of the protein and exhibits reduced sensitivity to Ca^2+^. Additionally, Anderson et al. found that the E2 component of KGDHC, DLST, is upregulated in T-cell acute lymphoblastic leukemia (T-ALL) (Anderson et al., 2016).

### Disease mechanisms underlying cycle enzyme alterations

Genetic alterations can occur in multiple cycle enzymes; however, their mechanisms of action in tumorigenesis differ. Both *SDH* and *FH* are classical tumor suppressor genes, and predispose individuals with heritable mutated genes to cancer when the second wild-type allele is lost (Chandel, 2015). Inactivating mutations in *FH* result in a build-up of fumarate and metabolic reprograming (Pollard et al., 2005), which includes an increased dependence on glycolysis and glutamine anaplerosis (Aspuria et al., 2014). In tumor cells harboring mutant *FH*, an accumulation of fumarate results in succination of cysteine-modifying proteins such as kelch-like ECH-associated protein 1 (KEAP1) and mitochondrial aconitase (ACO2) (Yang et al., 2014). Loss-of-function mutations of *SDH* result in the accumulation of millimolar concentrations of succinate and reduced levels of fumarate and malate (Pollard et al., 2005), which lead to disruption of multiple metabolic pathways including central carbon metabolism (Yang et al., 2013; Aspuria et al., 2014). On the other hand, *IDH1*/*2* missense mutations render the enzymes acquiring neomorphic activity that can convert α-KG to 2-HG (Dang et al., 2009). 2-HG is an oncometabolite that acts as a competitive inhibitor to α-KG-dependent dioxygenases, such as hypoxia-inducible factor (HIF), prolyl hydroxylases (PDHs), JmjC domain-containing histone demethylases, and ten-eleven translocation (TET) family of 5mC DNA hydroxylases (Chowdhury et al., 2011; Xu et al., 2011; Koivunen et al., 2012). The inhibition of these dioxygenases results in broad epigenomic alterations that both suppress differentiation and promote proliferation. Mutations in *IDH2*, *FH*, and *SDH* share a common mechanism of inhibiting α-KG-dependent dioxygenases through 2-HG, fumarate, or succinate, respectively (Hoekstra et al., 2015). Both *FH* and *SDH* mutations induce a state of pseudohypoxia, where 2-HG, fumarate or succinate can inhibit PHDs, resulting in stabilization of HIF. Additionally, mutations of *FH*, *SDH*, and *IDH1*/*2* cause increased production of reactive oxygen species (ROS), either directly by mutated *SDH* or indirectly in tumor cells with mutant *IDH1*/*2* and *FH* (Hoekstra et al., 2015). For example, glioma cells with *IDH* mutations have increased ROS and reduced GSH levels due to insufficient NADPH pools (Shi et al., 2015). In cancer cells with FH mutations, the accumulation of fumarate results in elevated levels of succinic-glutathione (GSF), which acts as an alternative substrate for GSH reductase, ultimately leading to decreased levels of NADPH and GSH (Sullivan et al., 2013).

## Potential approaches to target the TCA cycle

Therapeutically targeting the TCA cycle function in cancer is an attractive strategy to treat cancer and two strategies are currently being tested in the clinic. Many tumors utilize glutamine as a fuel source for the TCA cycle, thus suppression of glutaminolysis through small molecule inhibitors is an attractive approach to therapeutically target these tumors (Seltzer et al., 2010; Cheng et al., 2011; Le et al., 2012; Yuneva et al., 2012; Gameiro et al., 2013). An initial strategy utilized glutamine analogues, such as 6-diazo-5-oxo-L-norleucine, to target glutaminolysis (Ovejera et al., 1979; Ahluwalia et al., 1990; Griffiths et al., 1993). While these compounds highlight the potential of targeting glutamine anaplerosis, they ultimately failed to enter clinics due to high tissue toxicities. Additional studies have demonstrated that glutamine limitation, through either depletion of glutamine in the plasma (L-aspariginase) or blocking glutamine transport (sulfasalazine), can provide therapeutic benefit (Oettgen et al., 1967; Lo et al., 2008; Chan et al., 2014; Parmentier et al., 2015; Rodman et al., 2016; Roh et al., 2016; Shitara et al., 2017). Recently, GLS inhibitors, such as CB-839, an orally available, potent, and specific inhibitor of GLS, have shown anti-tumor efficacy. CB-839 disrupts the conversion of glutamine to glutamate and alters a number of downstream pathways, including the TCA cycle, glutathione production, and amino acid synthesis (Gross et al., 2010; Jacque et al., 2015). Phase I clinical trials are currently underway for CB-839, and examine its effectiveness for the treatment of both hematological malignancies and solid tumors (NCT02071927 and NCT02071888).

Besides targeting glutaminolysis outside the TCA cycle through GLS inhibition, several recent studies indicate that KGDHC represents a striking vulnerability for numerous cancers, and is a promising therapeutic target. Utilizing a MYC-driven model of T-ALL, Anderson and colleagues demonstrated that heterozygous inactivation of DLST (the E2 enzyme of KGDHC) was sufficient to significantly delay tumor onset without impacting normal animal development (Anderson et al., 2016). Additionally, they show that *DLST* inactivation in T-ALL cells disrupts the TCA cycle, while slowing cell growth and inducing apoptosis (Anderson et al., 2016). Allen et al. conducted a focused siRNA screen on TCA cycle enzymes, and found that many cancer cells highly depend on OGDH (the E1 component of KGDHC) for growth and survival (Allen et al., 2016). A recent study by Ilic et al. demonstrated that cancer cells harboring oncogenic *PI3K* mutations require all three components of KGDHC, *OGDH* in particular, for proliferation (Ilic et al., 2017). These findings support the rationale to target KGDHC for cancer treatment. CPI-613 is a lipoate analog that can simultaneously inhibit both PDH and KGDHC, as lipoate is a co-factor for both enzyme complexes. While CPI-613 stimulates PDK to phosphorylate and inactivate PDH (Zachar et al., 2011), CPI-613 can also induce a burst of mitochondria ROS through acting on DLD (the E3 component of KGDHC) and suppression of the E2 subunit of KGDHC, DLST (Stuart et al., 2014). Currently, CPI-613 is being tested in phase I and II clinical trials, as a single agent or in combination with standard chemotherapy, to treat cancers (NCT02168140, NCT01902381, NCT02232152, and NCT01766219). Limited data published from these trials have already shown that CPI-613 is generally well tolerated with minimal toxicity (Pardee et al., 2014; Lycan et al., 2016). While a phase I trial indicated that CPI-613 may be effective as a single agent for treating hematological malignancies, a phase II trial for small cell lung carcinoma show no efficacy as a single agent (Pardee et al., 2014; Lycan et al., 2016).

Finally, mutations in TCA cycle gene *IDH2* provide a unique opportunity for therapeutic intervention. Not only can mutant *IDH* serve as a biomarker, but also their neomorphic enzymatic activity can be targeted through small molecule inhibition. Currently, there are several small molecule inhibitors of mutant IDH2 in clinical development, including enasidenib (AG-221) that inhibits mutant IDH2 and AG-881 that targets both mutant IDH1 and IDH2 (Dang, 2016 #135). These compounds act by binding to the active catalytic site of mIDH1/2 enzymes and blocking the conformational change required to convert α-KG into 2-HG. AG-221 is an orally available inhibitor of mutant IDH2-R140 and IDH2-R172 (Yen et al., 2017), and is currently undergoing phase I/II clinical trials as a single agent for the treatment of AML and solid tumors (e.g., glioma and angioimmunoblastic T-cell lymphoma; NCT01915498 and NCT02273739, respectively). Preclinical data demonstrate that AG-221 can dramatically reduce 2-HG levels. Additionally, AG-221 results in cellular differentiation of tumor cells in murine xenograft models (Yen et al., 2017). Preliminary data from the AML clinical trial demonstrate that AG-221 alone led to a 41% object response rate and a 28% complete response rate. New phase I and III clinical trials will soon start and will examine the effectiveness of AG-221 alone in comparison to conventional therapy, as well as AG-221 in combination with standard induction and consolidation therapy (NCT02577406 and NCT02632708). The dual target inhibitor of mutant IDH1 and mutant IDH2, AG-881, is an orally available inhibitor that can pass the blood-brain barrier and may serve as a better option for glioma patients (Medeiros et al., 2017). Currently, AG-881 is in phase I clinical trial for AML patients with mutant IDH1/2, and a clinical trial for patients with glioma will begin soon (NCT02492737 and NCT02481154).

## Future perspectives

The TCA cycle is a critical metabolic pathway that allows mammalian cells to utilize glucose, amino acids, and fatty acids. The entry of these fuels into the cycle is carefully regulated to efficiently fulfill the cell’s bioenergetic, biosynthetic, and redox balance requirements. Multiple types of cancer are marked by drastic changes to TCA cycle enzymes, which result in characteristic metabolic and epigenetic changes that are correlated with disease transformation and progression. As a result, several components of the TCA cycle may be exploited therapeutically for the treatment of disease. However, due to the importance of the TCA cycle in normal cell development, high toxicity is a concern of this approach. Interestingly, although decreased KGDHC activity is associated with neurodegenerative diseases (Gibson et al., 2010), inhibiting KGDHC through CPI-613 is well tolerated in clinical testing (Pardee et al., 2014; Lycan et al., 2016). Additionally, 50% reduction of DLST, the E2 component of KGDHC, in zebrafish significantly delays MYC-driven leukemogenesis, without causing any detectable abnormalities (Anderson et al., 2016). Importantly, others show that cancer cells with IDH mutations become insensitive to treatment with mutant IDH inhibitors *in vivo*, owing to the metabolic rewiring and enhanced usage of the TCA cycle (Grassian et al., 2014; Tateishi et al., 2015). Emerging studies demonstrate that cancer cells utilize the TCA cycle differently from those of normal cells, making it likely that cancer cells will be more sensitive to inhibitors targeting the reprogrammed metabolic pathways in the TCA cycle (Kishton et al., 2016). These observations support the notion that targeting the TCA cycle by small molecule inhibitors of cycle enzymes and/or enzymes regulating the cycle could serve as a productive approach for cancer treatment.

Cancer cells often escape treatment through compensatory pathways (Obre and Rossignol, 2015; Zugazagoitia et al., 2016), and the metabolic properties of cancer cells are often context-dependent (Yuneva et al., 2012; Kishton et al., 2016; Martinez-Outschoorn et al., 2017). Hence, the key for successful metabolism-based therapies against cancer relies on both the identification of the “oncometabolic” enzyme(s) responsible for metabolic reprogramming and an in-depth understanding of the activity and flexibility of the altered pathways in the context of each specific cancer type. Despite the established role of the TCA cycle in tumorigenesis, its involvement in cancer metabolism remains incompletely understood. Additionally, how the TCA cycle interacts with other biochemical and cell signaling pathways is yet to be characterized. Owing to the impact of microenvironment on cellular metabolism and oncogenic signaling, it is critically important to study the contribution of the TCA cycle to cancer metabolism and tumorigenesis *in vivo*. Importantly, researchers started to successfully apply untargeted/targeted metabolomics and respiratory analyses to animal model organisms. The intensive research effort in the coming years will undoubtedly deepen our understanding of the role of this central metabolic hub that was once overlooked in tumorigenesis, reveal vulnerabilities for therapeutic intervention, and eventually bring this targeted approach from infancy up to maturity.
